# Delayed inhibition of an anticipatory action during motion extrapolation

**DOI:** 10.1186/1744-9081-6-22

**Published:** 2010-04-08

**Authors:** Welber Marinovic, Campbell S Reid, Annaliese M Plooy, Stephan Riek, James R Tresilian

**Affiliations:** 1 Perception & Motor Systems Laboratory, School of Human Movement Studies, The University of Queensland, Brisbane, Australia; 2 Department of Psychology, University of Warwick, Coventry, UK

## Abstract

**Background:**

Continuous visual information is important for movement initiation in a variety of motor tasks. However, even in the absence of visual information people are able to initiate their responses by using motion extrapolation processes. Initiation of actions based on these cognitive processes, however, can demand more attentional resources than that required in situations in which visual information is uninterrupted. In the experiment reported we sought to determine whether the absence of visual information would affect the latency to inhibit an anticipatory action.

**Methods:**

The participants performed an anticipatory timing task where they were instructed to move in synchrony with the arrival of a moving object at a determined contact point. On 50% of the trials, a stop sign appeared on the screen and it served as a signal for the participants to halt their movements. They performed the anticipatory task under two different viewing conditions: Full-View (uninterrupted) and Occluded-View (occlusion of the last 500 ms prior to the arrival at the contact point).

**Results:**

The results indicated that the absence of visual information prolonged the latency to suppress the anticipatory movement.

**Conclusion:**

We suggest that the absence of visual information requires additional cortical processing that creates competing demand for neural resources. Reduced neural resources potentially causes increased reaction time to the inhibitory input or increased time estimation variability, which in combination would account for prolonged latency.

## Background

An important topic in neuroscience is to understand how visual information is employed to guide goal-directed actions. An influential proposal regarding the use of visual information to control purposeful actions was put forward by Lee [1,2]. According to Lee's proposal, the inverse of the relative rate of image size change of an approaching object constitutes a first-order two-dimensional source of information, referred to as *tau*, which can be used to specify time-to-contact. Although the utility of Lee's original proposal has been called into question a number of times [3-5], the idea that time-to-contact information derived from the optical flow can be used for the timing of interceptive actions attracted much attention to the investigation of this type of motor action ([6,7] for reviews). Influenced by Lee's proposal (see also [8]), most models for the control of anticipatory actions (e.g. hitting or catching) suppose that descending motor command generation is initiated when time-to-contact reaches a criterion value [7,9]. Note that this triggering process can be initiated by a variety of perceptual variables other than *tau *and which could also be employed for timing interceptive actions in conditions other than direct approach towards the eyes [4,10-12]. Although in an ideal scenario visual information is available continuously, there are instances in which the objects with which people must interact are occluded by other (static or moving) objects in the environment. For example, a keeper in soccer must judge when a ball will arrive at the goal, so that he can stop it from going in, while having his vision occluded by other players (teammates and opponents) in the field. Nonetheless, even when continuous visual information is unavailable people appear to be able to use a signal derived from motion extrapolation or temporal estimation processes to initiate their actions [13,14]. These time estimation processes are likely to be aided by the activity of specific areas in the cerebellum which are believed to reflect the operation of internal models based on memory of the previous motion of moving objects [15,16].

The cerebellar activity involved in time estimation may represent an additional processing step, which also functionally connects with the fronto-parietal attentional network as well as sensory-motor networks [16]. This additional processing may have an effect on the overall processing time, but it is also likely to be affected by the attentional demands of the task. Dual-task paradigms have been extensively employed to investigate the role of attention in temporal estimation processes (see e.g. [17-20]). Overall, the results of these experiments demonstrate that there is an interference effect, which is reflected in a reduction in temporal estimation accuracy when a secondary task diverts attention from the timing task [17]. These results have been interpreted under the perspective that we possess a limited pool of attentional resources that must be shared in case other cognitive processes are required to overlap at some point in time with the time estimation process (e.g. [21]). Thus, when a cognitive process, other than the time estimation process, must be initiated whilst a temporal interval is being estimated, attentional resources must be allocated to it and the accuracy of the timing process is then impaired.

The studies investigating the effects of attentional overload (secondary task) over the timing task (primary task) usually required that the participants performed different tasks both simultaneously and in isolation. Temporal estimation accuracy is assessed by measuring the produced intervals under the different experimental conditions (single vs. dual-task). In this article, we also sought to tackle the role of attention in performance but from a different perspective. More specifically, we sought to determine whether the requirement to accurately predict the initiation of an anticipatory motor response through temporal estimation processes could affect the minimum time required to inhibit an anticipatory action. Thus, rather than the accuracy of the temporal estimation process itself, our interest in the present report was to determine whether the use of temporal estimation processes to time movement initiation would interfere with the ability to attend to a sudden change in the goal of the task. Although the inhibition of anticipatory actions has been previously studied in the motor control literature [22-25], to our knowledge no study has been conducted to explore the impact of availability of visual information on the time needed to inhibit an anticipatory timing action.

**Figure 1:**
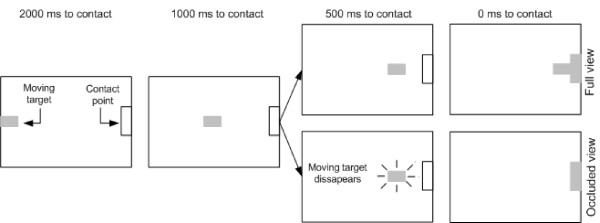
**Schematic representation of the time course of events in the full view and occluded view blocks**.

In the experiment reported here, we examined the ability to inhibit an anticipatory task in two different conditions of visual stimulus: a) full view (continuous visual information as shown in Figure 1) and b) occluded view (occlusion of the final part of the moving object's trajectory as shown in Figure 1). The aim of the experiment reported was to determine whether or not the employment of a cognitive temporal estimation process to time an interval in the hundreds of milliseconds range could affect the latency to inhibit an anticipatory action.

## Methods

### Participants

Eight volunteers participated in the experiment and all gave their informed consent prior to commencement of the study, which was approved by the local Ethics Committee of the University of Queensland. All participants reported normal or corrected to normal vision and stated they were right handed. Their ages ranged from 22 to 36 years (mean = 28.75 years).

### Apparatus and task

The primary task was to abduct their first index finger against a force transducer in synchrony with the arrival of a moving object (50 × 25 pixels) at a determined contact point (25 × 50 pixels) on a monitor screen. However, on 50% of the trials, when a red stop sign (stop-signal) appeared on the screen (210 × 210 pixels), the participants were required to halt their movements. The stop-signal appeared pseudo-randomly on the screen (next to the contact point) at various times (82, 117, 152, 188, 223, and 258 ms) prior to the arrival of the moving object at the contact point. The stop-signal remained on the screen until 500 ms after the arrival of the moving object at the contact point. The moving object took 2 seconds to travel from the left to the right side of a 19" monitor screen (85 Hz refresh rate, 1024 × 768 resolution) located 0.9 meters away from the participants. All visual stimuli were presented using Cogent 2000 Graphics running in MATLAB 7.0.

### Design and procedures

The experiment was run in two blocks: *a*) full view, and *b*) occluded view. In the occluded view condition the moving object disappeared from view 500 milliseconds before its arrival at the contact point as shown in Figure 1. During practice the participants performed 50 trials without stop-signals to learn the correct time of movement onset. An additional 24 practice trials were provided, during which each stop-signal was pseudo-randomly presented twice. Practice was provided for both full and occluded view. Following practice, half of the participants began the experiment with block *a*, and half with block *b*. The participants performed 10 trials for each of the 6 conditions in which there was a stop-signal plus 60 control trials (no stop-signal) in each of the two blocks (240 total). Feedback about the temporal error was presented after control trials to encourage the participants to achieve optimal performance. Feedback about whether or not the participants succeeded to inhibit their movements was provided after stop-signal trials to motivate the participants to attend to the stop-signal.

### Analysis

The variable of interest was the absence or presence of movement. A failure to withhold a movement was considered as such if the participant reached a torque level which exceeded 3 standard deviations from baseline levels for more than 40 samples. The torque transducer data was sampled at 2000 Hz.

Since the percentage of successful inhibitions follows a binomial distribution, we used the arcsine squared root transformation to analyse this variable as recommended by Hogg and Craig [26]. The mean transformed percentage of successful inhibitions were submitted to separate 2 (view condition: FV and OV) × 6 (stop-signal interval: 82, 117, 152, 188, 223, 258 ms) repeated measures analysis of variance. A Newman-Keuls post-hoc test, p < .05, was conducted to determine the locus of significant differences involving more than two means when the corresponding analysis of variance was significant.

Additionally, we also used a paired t-test to analyze the constant temporal error obtained in control trials (no stop-signal) in FV and OV. This analysis served to verify whether the participants used a specific strategy such as delaying movement onset to deal with the absence or not of visual information. Note that because of the nature of anticipatory actions (where the participants choose when to start their movements), there is no reaction time to be measured and, therefore, the constant temporal error indicates whether or not the participants systematically delayed their responses in a particular condition (see [23,25]). Note that delaying the time of movement onset in our task would increase the chances of successfully inhibiting responses as the corrected stop-signal interval would be greater than expected had the participants initiated their actions at the correct time. However, delaying the time of movement in the control trials would result in an observed delay in the temporal error, which was the sort of behavior we sought to discourage by giving feedback as mentioned previously.

## Results

**Figure 2:**
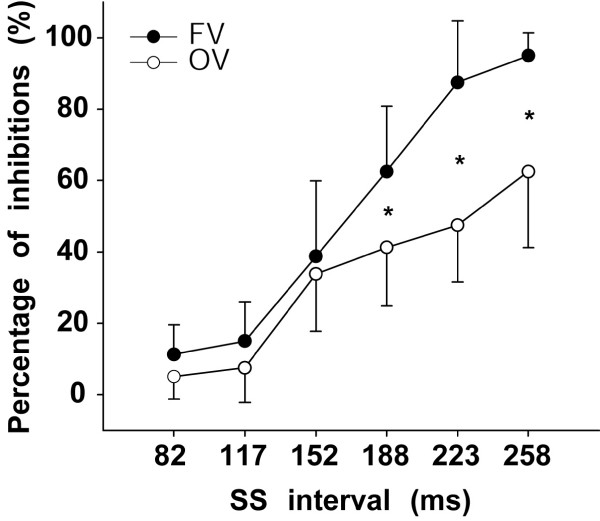
**Mean percentage (non-transformed) of successfully inhibited responses as a function of the stop-signal (SS) interval**. Black circles represent the full view block (FV). White circles represent the occluded view block (OV). * Marks significant contrasts between FV and OV. Error bars represent 95% confidence intervals.

The repeated measures ANOVA on the transformed percentage of successfully inhibited trials showed a reliable main effect of viewing condition, F_(1,7) _= 8.94, p < .05, η_p_^2 ^= .56. This main effect shows that the participants were able to inhibit more movements in the full view condition than in the occluded view condition, as we predicted. There was also a reliable main effect of stop-signal interval, F_(5,35) _= 56.18, p < .001, η_p_^2 ^= .89. The post-hoc test of this effect revealed, as expected, that the success to inhibit in both conditions increased as the interval available to halt the actions became longer. More importantly, The analysis of variance also showed that the interaction between viewing condition and stop-signal interval was statistically significant, F_(5,35) _= 4.81, p < .01, η_p_^2 ^= .41. Post-hoc pairwise comparisons of this interaction showed significant differences between FV and OV at stop-signal intervals of 188, 223, and 258 ms, as shown in Figure 2. This indicates that the participants suppressed significantly more responses between 258 and 188 ms prior to the arrival of the moving stimulus at the contact point in the full view condition than in the occluded view condition. At SS intervals shorter than 188 ms there was no significant difference in the number of responses suppressed in the two conditions (FV *vs*. OV).

In order to verify whether the interaction between viewing condition and time was not simply due to a strategy employed by the participants to delay movement onset in the full view condition, we compared the constant temporal error observed in control trials in FV and OV. The mean constant temporal error (± SD) in the full view condition was 11.0 ms (± 23.0), whereas in the occluded view condition it was 5.1 ms (± 28.5). A paired t test failed to show a significant difference between these means, *t*(7) = .41, p > .05, r = .15. This seems to suggest that the differences found in the percentage of responses inhibited were not due to a bias for the participants to hold their responses in the full view condition. In addition, to be certain of our assertion that the participants did not systematically delayed their responses in the FV condition, we also fitted the non-transformed probability of inhibiting the response for each time at which the stop-signal was presented with a cumulative Gaussian by using a maximum-likelihood fitting procedure. The mean time required to suppress the action was defined as the point in the inhibition function at which the probability of inhibiting the movement was 0.5. Following Slater-Hammel procedures [25], the time to inhibit was then corrected for each participant based on the constant temporal error obtained in control trials (trials without a stop-signal).

The mean (± SD) corrected time to inhibit a response in the full view block across participants was 176.4 ms (± 17.4), whereas in the occluded view block the mean was 234.8 ms (± 32.2). A paired t test comparing the means in each condition showed that the difference between means was statistically significant, *t*(7) = 6.97, p < .001, r = .93. This result, therefore, supports our initial analysis of the percentage of successfully inhibited trials and indicates that the manipulation of visual information can affect the minimum time required to suppress an anticipatory action.

## Discussion

The results of the experiment clearly showed that the minimum time required to suppress an anticipatory timing task was prolonged when the participants were forced to use time estimation processes to initiate their responses. The longer minimum suppression time for the prediction-motion task (occluded view) was the result expected if greater attentional resources had to be deployed to time movement onset, reducing the resources available for responding to the stop-signal. Competing for attentional resources could increase stop-signal reaction time or decrease the accuracy of the temporal estimation process. It is likely that a combination of these two effects leads to the longer minimum suppression time.

One could have expected that there would be no difference in the minimum time required for movement inhibition in our experiment for two reasons. Firstly, previous studies investigating performance in prediction-motion tasks showed that performance suffers more only when the interval to be estimated is longer than 1 second [27,28]. This could indicate that timing an interval of 500 milliseconds would not represent a challenge to the participants and their performance to inhibit the action could be similar in both experimental conditions. Secondly, Lewis and Miall [29] propose that timing in the hundreds of milliseconds range is automatic and therefore does not depend upon neural systems associated with attention and working memory. If this estimation process could be entirely automatic in our task one would not expect significant differences in the minimum time required for suppression.

It has been suggested that prediction-motion tasks (occluded view conditions) involve cognitive processes of motion extrapolation [14], whereas interceptions and coincidence-anticipation tasks with full view do not [30]. This suggests that prediction-motion tasks involve the so-called cognitive visual system that involves the ventral stream of visual processing [31,32], whereas the full-view tasks involve the motor visual system (dorsal stream of processing) that operates without conscious or cognitive processing [33]. There is a body of data consistent with this proposal [30]. In the full view condition, information about the target is accessed directly through visual input via the dorsal stream. If the response to the stop-signal is processed via the ventral stream then the two information streams are kept separate, minimizing attentional load. In the occluded view condition, information about the target is internally generated via a system that interacts with the cerebellum, putamen and the fronto-parietal attentional network [16]. This leads to the possibility that the temporal estimation and inhibition response are competing for resources within the fronto-parietal network, reducing the efficacy of both. This would be expected to increase the time taken to respond to the stop-signal in the occluded view condition relative to the full view condition. Note that since this competition for attentional resources is likely to be caused in great part by the stop-signal requiring processing in the ventral visual stream, it is uncertain whether the same results would hold if an auditory stop-signal was used.

Alternatively, the additional time required to inhibit anticipatory actions during occlusions could result from interference at the cerebellar level. Recently, Ghajar and Ivry [34,35] have suggested that the cerebellum is part of an anticipatory network that also engages the prefrontal cortex and the parietal lobe. According to these authors [34,35], the cerebellum can reduce performance variability not only for motor actions, but also for higher-order processes by temporally coordinating the interactions between prefrontal cortex and temporal lobe. If the initiation of anticipatory actions during occlusion requires the cerebellum to produce a model of the motion of a previously seen moving object [15,16], at the same time at which it is involved in synchronizing the activity of the prefrontal cortex and the parietal lobe, one could reason that interference is established at this level. The role of the cerebellum proposed by Ghajar and Ivry [34,35] is supported by data showing that patients with cerebellar lesions demonstrate impaired performance in tasks requiring shifts of attention [36,37].

Also interesting in our results is the significant interaction between viewing condition (FV vs. OV) and time of stop-signal presentation in the percentage of inhibited responses. As shown in Figure 2, the probabilities of inhibiting responses at short stop-signal intervals (≤ 152 ms) were below 50% in both viewing conditions. After 188 ms prior to movement onset, however, the percentage of responses inhibited in the full-view condition increased more sharply than in the occluded-view condition. The low percentage of inhibited responses in both conditions at short stop-signal intervals is consistent with the triggering of descending motor commands (see Introduction) occurring about 150 ms prior to movement onset as recently reported for fast interceptive actions in conditions where no inhibition of the movement was required [38,39]. In other words, the two viewing conditions did not differ much for short stop-signal intervals because at these times the inhibition process triggered by the stop-signal (see [23]) could not be finished before the descending motor commands had already been released. However, for earlier presentations of the stop-signal (>152 ms), there was a greater chance that the inhibition process could at least start before descending motor commands had been triggered. It was for these longer presentations of the stop-signal (≥ 188 ms) that the differences between full and occluded view conditions began to accrue.

## Limitations

We should highlight that our findings were obtained with an anticipatory task where time-to-contact judgments were based upon the observation of targets moving laterally in relation to the participants (left-to-right on the monitor screen). Human MRI data has shown that time-to-contact prediction during fronto-parallel displacement of moving targets activates cortical areas such as the superior parietal sulcus and the marginal ramus of the cingulate sulcus, which are areas not activated during looming approach (e.g. head-on collisions) [40]. Since different processing systems could be involved in determining time-to-contact for different types of approach (lateral *vs*. looming), one should be cautious of generalising our results to other forms of approach. Further research should investigate whether our results can be extrapolated for looming approach and whether motion extrapolation can cause interference when the processing of the stop-signal is not carried out by the visual ventral system.

## Conclusion

In summary, the results demonstrated that the manipulation of visual information can significantly affect the minimum time needed to inhibit movement in an anticipatory timing task. From a cognitive perspective this effect could be due to the engagement of distinct visual systems (motor vs. cognitive) to initiate motor responses inducing different attentional loads and, as a result, affecting the latencies required to suppress an anticipatory action. From a neurophysiological perspective interference at the cerebellar level could prevent the participants from readily attending to the stop sign and increase the stop-signal reaction time. These two accounts are not mutually exclusive and further experiments are warranted to examine this phenomenon.

## Competing interests

The authors declare that they have no competing interests.

## Authors' contributions

WM designed, collected the data, analyzed and drafted the manuscript. CR participated in the data analysis and contributed to the interpretation of the results. AMP and SR participated in the acquisition of data, discussions about the data analyses and commented on the written drafts of the manuscript. JRT participated in the design and interpretation of the results. All authors have read and approved the final manuscript.
